# GLUT1 and prorenin receptor mediate differential regulation of TGF-β and CTGF in renal inner medullary collecting duct cells during high glucose conditions

**DOI:** 10.1186/s40659-024-00560-8

**Published:** 2024-11-07

**Authors:** Paulina E. Larenas, Pilar Cárdenas, Monserrat Aguirre-Delgadillo, Carlos Moris, Dulce E. Casarini, Zoe Vallotton, Minolfa C. Prieto, Alexis A. Gonzalez

**Affiliations:** 1 Instituto de Química, Pontificia Universidad Católica de Valparaíso, Valparaíso, Chile; 2 Departamento de Medicina, Disciplina de Nefrología, Escola Paulista de Medicina, Universidade Federal de São Paulo, São Paulo, Brazil; 3 Department of Physiology and Tulane Hypertension and Renal Center of Excellence, Tulane University School of Medicine, New Orleans, LA USA

**Keywords:** Diabetes, Kidney, Renal fibrosis, Prorenin, High glucose

## Abstract

**Background:**

During diabetes, prorenin is highly produced by the renal collecting ducts. The binding of prorenin to (pro)renin receptor (PRR) on the apical plasma membrane triggers intracellular profibrotic genes, including TGF-β and CTGF. However, the underlying mechanisms contributing to the stimulation of these pathways remain unclear. Hence, we hypothesize that the glucose transporter-1 (GLUT1) favors the PRR-dependent stimulation of TGF-β and CTGF in the distal nephron segments during high glucose (HG) conditions.

**Methods:**

To test this hypothesis, primary cultured renal inner medullary collecting duct (IMCD) cells were treated with normal glucose (NG, 5 mM) or high glucose (HG, 25 mM) for 48 h in the presence or absence of the GLUT1-specific inhibitor BAY 876 (2 nM). Additionally, IMCD cells were treated with the PRR antagonist PRO20. The expression of TGF-β and CTGF was quantified by immunoblot and qRT-PCR.

**Results:**

HG increased GLUT1 mRNA and protein abundance, while BAY 876 inhibited these responses. HG treatment upregulated PRR, but the concomitant treatment with BAY 876 partially prevented this effect. TGF-β and CTGF expressions were augmented in IMCD cells treated with HG. However, PRO20 prevented the increases in TGF-β but not those of CTGF. GLUT1 inhibition partially prevented the increases in reactive oxygen species (ROS) during HG while PRO20 did not. ROS scavenging impaired CTGF upregulation during HG conditions. Additionally, long-term exposure to HG increases lipid peroxidation and reduced cell viability.

**Conclusions:**

The data indicate that glucose transportation via GLUT1 is implicated in the PRR-dependent upregulation of TGF-β while CTGF is mediated mainly via a mechanism depending on ROS formation in renal medullary collecting duct cells.

**Supplementary Information:**

The online version contains supplementary material available at 10.1186/s40659-024-00560-8.

## Introduction

In diabetes mellitus (DM), there is activation of the intrarenal renin‐angiotensin system (RAS) through the renal uptake of prorenin and angiotensinogen (AGT), increased Angiotensin-converting enzyme (ACE) immunostaining in tubular and interstitial cells, elevated levels of angiotensin II (Ang II), and enhanced intrarenal AGT mRNA and/or protein levels [1–3]. Combined actions from renin, prorenin, and (pro)renin receptor (PRR) contribute to the activation of the intratubular RAS, tubular interstitial damage [4–8], and diabetic nephropathy [4, 5, 9, 10]. The PRR is considered a novel member of the RAS able to bind prorenin and renin, thus increasing the formation of angiotensin (Ang) I and subsequently Ang II [11, 12]. Prorenin and renin binding to the PRR also triggers intracellular profibrotic responses [13–15] and kidney injury [16], independent of Ang II formation [13, 14, 17].

Among the injury markers observed in chronic DM in experimental animal models, the profibrotic factors transforming growth factor-beta 1 (TGF-β1), fibronectin, and connective tissue growth factor (CTGF) have been found to be mostly responsible for the profibrotic phenotype in tubular cells, which enhances the proliferation of fibroblasts and collagen deposition [18]. Tubular markers of injury in diabetic disease are associated with proteinuria and albuminuria, however the progression of tubule-interstitial disease in addition to glomerular injury in diabetes is also important and may provide insights into the pathogenesis of diabetic nephropathy beyond the glomerular injury. Evidence indicates that high glucose increases TGF-β and fibronectin expression in mouse inner medullary collecting duct (IMCD) cells via NOX-4-dependent production of reactive oxygen species (ROS) [19]. Additionally, PRR activation stimulates ROS in several tissues [20–22]. This supports the concept that during HG conditions, PRR cooperates to augment fibrosis and renal injury intracellular signaling.

Most of the cells in the body exposed to high glucose show a decreased rate of glucose transport into the tissues caused by reducing GLUT1 mRNA and protein expression, as well as GLUT1 plasma membrane localization. Under normal physiological conditions, the kidney is able to filter large amounts of glucose by complete reabsorption in the proximal tubule. During pathophysiological conditions such as diabetic induced hyperglycemia, the excessive filtration of glucose and impaired uptake in the proximal tubule promote higher amounts of glucose that reach the distal nephron [23]. In contrast to what is observed in peripheral tissues, GLUT1 expression in tubular segments is increased in models of activation of RAS and diabetic rats [24]. Furthermore, a correlation has been shown between GLUT1 abundance and the glycolytic activity of collecting duct segment. Therefore, it is suggested that distal tubule segments take up glucose as a source of energy via GLUT1 [25]. In rat kidneys, GLUT1 is abundantly expressed in collecting ducts, especially in principal cells and in intercalated cells [26]. Intercalated cells also express PRR, which has been shown to be upregulated by high glucose conditions in several reports [8, 27, 28].

High glucose also increases PRR trafficking to the plasma membrane of collecting duct cells in male streptozotocin (STZ)-Sprague–Dawley hyperglycemic rats [8]. Prorenin is the natural agonist of PRR and is highly produced and secreted by the collecting duct in rodent models of hyperglycemia and diabetes [7]. Thus, coordinated actions between GLUT1 and PRR during HG conditions may contribute to the activation of profibrotic pathways. However, the mechanisms are not clearly understood. To elucidate the specific role of PRR in this process, pharmacological agents have been generated, including the Decoy Peptide HPR [29, 30] which inhibits diabetic nephropathy in diabetic rat models [31]. More recently a 20-aminoacid peptide (PRO20) consisting of the prorenin pro-segment has been created and its actions confirmed in several studies [32–34]. In the present study, we aimed to examine the cooperative roles of PRR and GLUT1 on the stimulation of TGF-β and CTGF signaling during HG conditions in cultured renal IMCD cells using GLUT1 inhibition with BAY 876 and PRR blockade with PRO20, the pharmacological antagonist of PRR.

## Results

### Primary cultures of mouse inner medullary collecting ducts (IMCD) cells

**Fig. 1:**
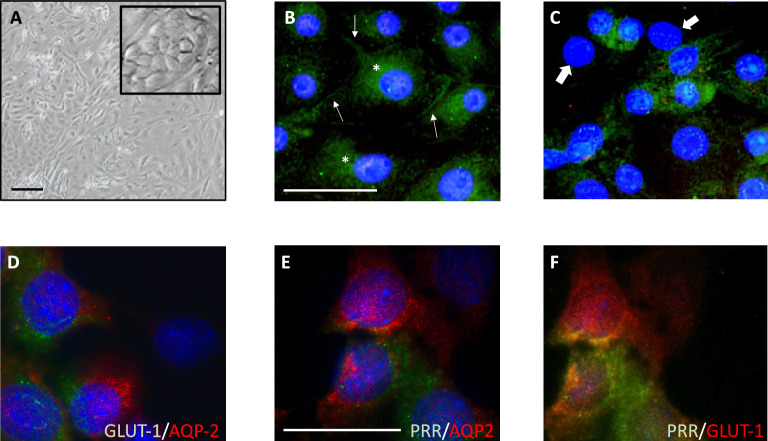
Characterization of mouse inner medullary collecting ducts (IMCD)**.** IMCD cells grown in NG conditions and then treated with HG. **A** Light field microscopy image shows the characteristic shape and phenotype of the primary cultured IMCD cells (Scale bar: 100 μm). The zoom in image shows the cuboidal characteristic shape of primary cultures of IMCD cells after 6 d. **B** IMCD were characterized by the expression of collecting duct marker AQP-2 (green) in the plasma membrane (arrows) and cytoplasm (asterisk) of the principal cells. **C** IMCD cells were stained with PRR (green) showing some PRR-positive cells as well as negative cells (wide arrows) which are mainly principal cells. Scale bar for **B** and **C**: 20 μm. Co-localization experiments further demonstrate no colocalization of AQP-2 with GLUT1 (**D**) or PRR (**E**). However, GLUT1 colocalized with PRR (**F**). Staining of nucleus with DAPI. Scale bar for D, E and F: 20 μm. Immunofluorescence and phase contrast images were obtained using a Nikon Eclipse-50i immunofluorescence microscope (Nikon Eclipse-50i, Japan) and were digitalized using the NIS-Elements version 4.0 from Nikon

Primary cultures of male mouse IMCD cells were incubated under normal (5 mM) or high (25 mM) glucose conditions. Before HG treatment, cell growth and confluence were evaluated under a microscope using a minimum of 15 fields and 3 random wells. Figure S1A shows cell and tissue adhesion after 12 h, 2, 3, and 6 d. After three days, scattered groups of cells were growing. Cell cultures on day six of growth before treatment showed 70–80% confluence. HG was then applied and evaluated following 48 h of treatment. Figure S1B shows no effect on cell number per field after adding NG, HG, or the osmotic control Mannitol. (Control: 242 ± 12 vs. HG: 231 ± 34 vs. Mannitol 25 mM: 263 ± 51 cells per field, Fig. 1SB). Figure 1A shows a representative field after 80–90% confluence. Characterization of the presence of aquaporin-2 (AQP-2) and the (pro)renin receptor (PRR) in principal and intercalated cells, respectively, is shown in Fig. 1B and C. AQP-2 was found in plasma membrane (Fig. 1B, arrows) and also in the cytoplasm (Fig. 1B, asterisks). In Fig. 1C we shown that PRR is present in intercalated cells and is depicted in green (wide-white arrows indicate cells with negative staining for PRR, likely principal cells). To further confirm the specific expression of GLUT1, we performed co-staining immunofluorescence. Figure 1D, E and C show that GLUT1 and AQP-2 does not colocalize in the same type of cells. PRR and GLUT1 showed yellow-color indicating colocalization of both proteins. Antibody specificity was performed by omission of the primary antibody (Figure S1C). This data suggests that GLUT1 was present mostly in intercalated cells which agrees with previous report [26].

### High glucose increases GLUT1 mRNA and protein expression in IMCD cells

**Fig. 2:**
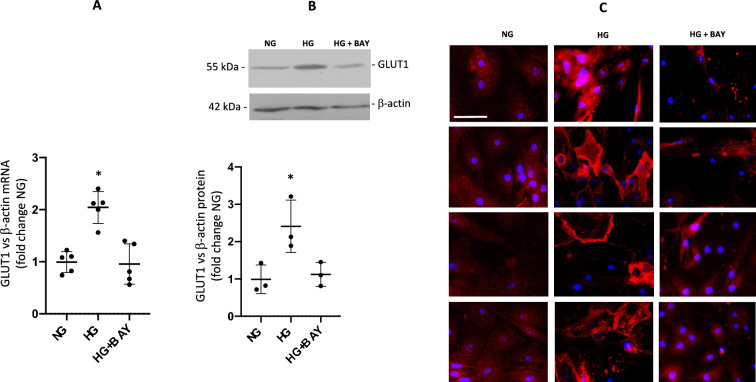
GLUT1 expression following 48 h conditioning with NG, HG, or HG + GLUT1 inhibitor BAY 876 pretreatment. **A** mRNA levels of GLUT1 under treatment with HG alone were augmented almost twice that of controls. GLUT1 inhibition by BAY 876 (2 nM) for 48 h prevented the upregulation of GLUT1 (*p < 0.05, n = 5). **B** Similar results were observed when analyzing protein levels (*p < 0.05, n = 3). **C** Four representative immunofluorescence fields showing that GLUT1 abundance (red) was augmented after 48 h HG treatment, while HG + BAY 876 prevented this effect (nuclei in blue). Scale bar represents 20 µm

GLUT1 has been detected in medullary and cortical collecting ducts [24, 35, 36]. We evaluated the effect of 48 h of HG treatment on GLUT1 expression. mRNA levels of GLUT1 were augmented almost twice that of controls (fold change control: 2.1 ± 0.3 vs. 1.0 ± 0.2, p < 0.05, Fig. 2A). Similar results were observed when analyzing protein levels (fold change control: 2.4 ± 0.7 vs. 1.0 ± 0.4, p < 0.05, Fig. 2B). Immunofluorescence suggested that GLUT1 abundance was augmented most likely in the plasma membrane (fluorescence intensity pixels per field: 3,562 ± 35 vs. 1,652 ± 113, p < 0.05, Fig. 2C). Interestingly, incubations with the GLUT1-specific inhibitor BAY 876 (2 nM) before HG prevented these effects.

### High glucose increases prorenin and renin secretion, PRR expression, and ERK 1/2 phosphorylation, while GLUT1 inhibition impairs these effects

**Fig. 3:**
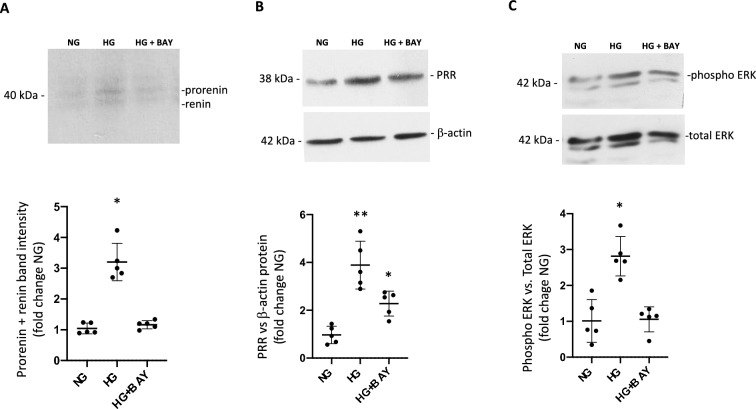
HG conditions stimulate prorenin and renin release to the cell culture media, PRR upregulation, and activation of MAPK pathway; GLUT1 inhibition prevented these increases. **A** Prorenin and renin band detected by immunoblot in concentrated cell culture media. Intensity of prorenin-renin bands (together) was augmented in cell culture media in HG conditions but was prevented in cells pretreated with BAY 876. 40 μg total protein were loaded in each lane. **B** PRR was augmented in cell lysates of IMCD cells under both HG and HG + BAY 876 conditions, however, incubation with BAY 876 mitigated the extent of the response. **C** Phospho-ERK1/2 versus total ERK1/2 ratio was augmented after 48 h of HG; BAY 876 pretreatment impaired this effect. *p < 0.05 vs NG; **p < 0.01 vs NG

Previous studies have shown that HG can increase prorenin in collecting duct cells [7, 37–39], but it is unknown whether this phenomenon depends on the GLUT1-mediated transport of glucose. We tested the effect of HG conditions on prorenin-renin secretion using immunoblot detection of both bands from cell culture media as previously described [30]. Figure 3A shows prorenin and renin bands detected by immunoblot. As observed, HG alone increased prorenin-renin band abundance in cell culture media (3.2 ± 0.6 vs 1.0 ± 0.2, p < 0.05), however, this effect was prevented in cells previously incubated with BAY 876 (1.2 ± 0.1 vs 1.0 ± 0.2, p = non-significant). We next tested the abundance of PRR in cells treated with HG alone or in conjunction with cells pretreated with BAY 876. While HG alone increased PRR protein abundance (3.9 ± 1.0 vs 1.0 ± 0.3, p < 0.05), pretreatment with BAY 876 partially prevented this increase (HG vs HG + BAY 876: 3.9 ± 1.0 vs 2.2 ± 0.5, p < 0.05, Fig. 3B). Since PRR triggers intracellular MAPK pathways [40], we tested the effect of GLUT1 inhibition on the phosphorylation of ERK1/2 (Fig. 3C). We found that HG treatment for 48 h augmented the ratio of phospho-ERK1/2 versus total ERK1/2 (fold change of control: 2.8 ± 0.5 vs. 1.0 ± 0.5, p < 0.05); HG + BAY 876 partially prevented this effect (fold change of control: 1.1 ± 0.3 vs. 1.0 ± 0.5, p = non-significant).

### High glucose increases TGF-β and CTGF expression while GLUT1 inhibition impairs this effect

**Fig. 4:**
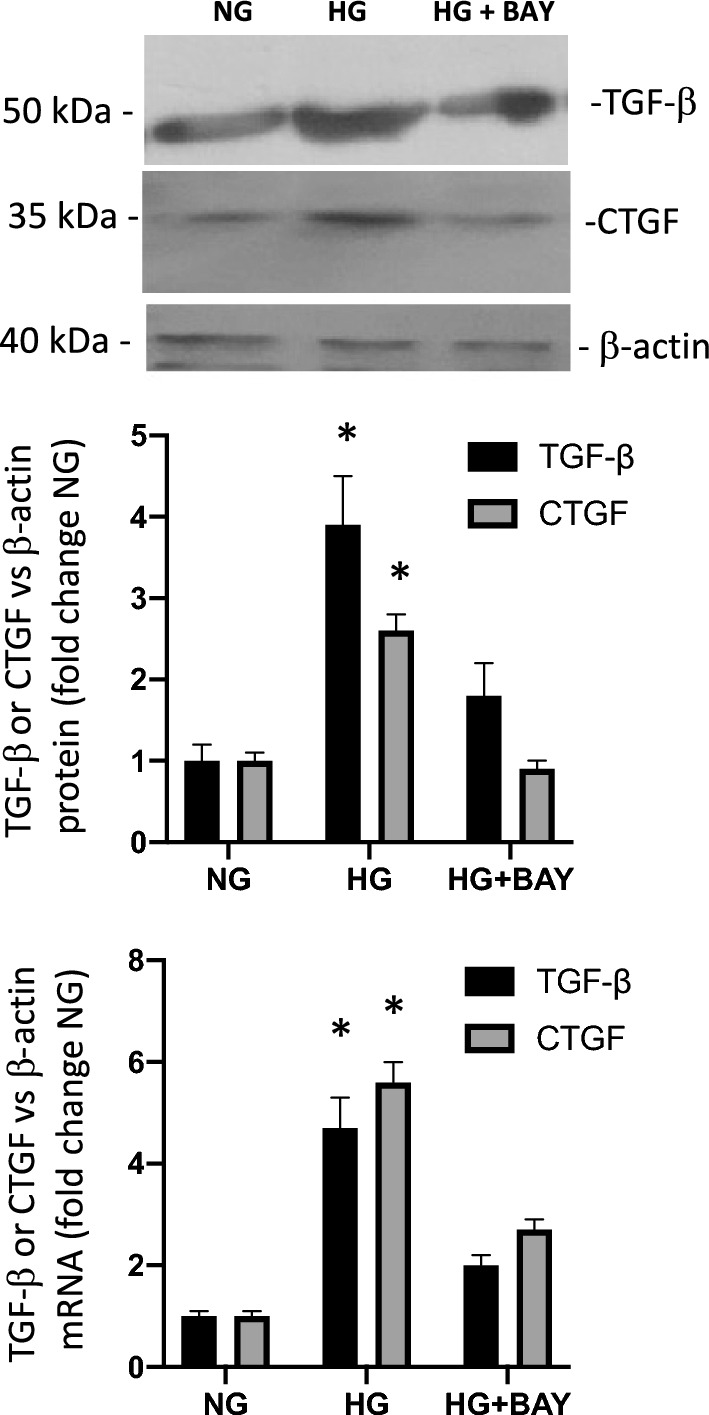
IMCD cell protein and mRNA levels of TGF-β and CTGF following high glucose treatment and GLUT1 blockade. Immunoblot of IMCD cells measuring TGF-β and CTGF protein expression under the conditions of NG, HG, and HG plus the GLUT1 inhibitor BAY 876 for 48 h (HG + BAY) is shown along with fold change of treatment compared to NG expressed as the ratio of protein to housekeeping gene β-actin. The bottom-most plot shows the mRNA abundance as fold change of treatment compared to NG. mRNA levels were normalized using housekeeping gene β -actin. No effect was observed in NG + BAY 876 (not shown). *p < 0.05 versus NG, n = 3–4

In rat and mouse models of diabetes, both TGF-β and CTGF are increased in renal collecting ducts [41, 42]. We and others have demonstrated that some of the mechanisms behind this increase are directly mediated by the activation of PRR [14, 15, 43]. Thus, we evaluated if the inhibition of GLUT1 could prevent the induction of TGF-β and CTGF in cultured IMCD cells. Figure 4 shows the expression of TGF-β and CTGF in cultured IMCD after 48 h of HG alone or in combination with IMCD cells previously treated with BAY 876. HG alone resulted in increased abundance of TGF-β and CTGF proteins (TGF-β: 3.9 ± 0.6 vs. control 1.0 ± 0.2, p < 0.05; CTGF: 2.6 ± 0.3 vs. control 1.0 ± 0.2, p < 0.05) and mRNA levels (4.7 ± 1.3 vs. control 1.0 ± 0.3, p < 0.05; CTGF: 5.6 ± 0.9 vs. 1.0 ± 0.3, p < 0.05). GLUT1 inhibition with BAY 876 under HG conditioning prevented this increase showing no statistical differences with the control group. However, CTGF mRNA expression was significantly higher than control but reduced under HG alone (HG + BAY 876: 2.7 ± 0.5 vs. control 1.0 ± 0.3, p < 0.05).

### High glucose increases TGF-β and CTGF expression while PRR blockade impairs the upregulation of TGF-β but not CTGF

**Fig. 5:**
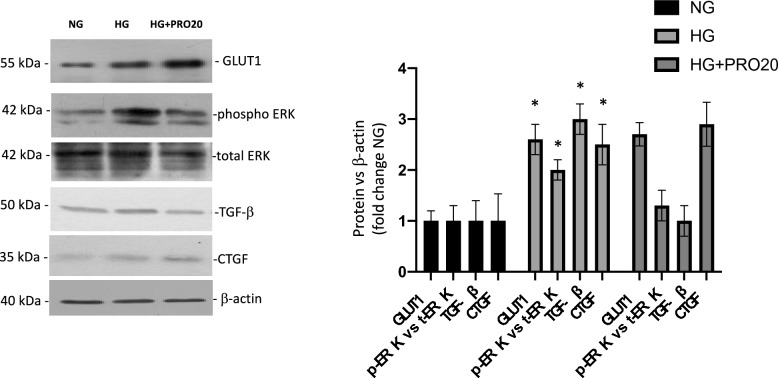
PRO20 prevented the induction of TGF-β but not CTGF after 48 h of HG treatment in IMCD cells. Protein and abundance levels of GLUT1, phosphorylated ERK, total ERK, TGF-β and CTGF during NG, HG, and HG + PRO20 conditions. Representative immunoblot (left) and protein abundances (right) quantified as the ratio protein versus β-actin. GLUT1, phosho ERK, CTGF and TGF-β abundances were augmented in HG conditions. Preconditioning with PRO20 prevented ERK phosphorylation and upregulation of TGF-β but not CTGF. No effect was observed in NG + PRO20 (not shown). *p < 0.05 versus NG, n = 3–4

Previous research has shown that prorenin treatment in M1 collecting duct cell lines increases TGF-β and CTGF through the MAPK pathway [15]. First, we evaluated the possibility that GLUT1 protein and mRNA levels may change in conditions of HG and PRR blockade. As shown in Fig. 5, PRR blockade does not affect GLUT1 protein or mRNA levels. Since it is unclear whether TGF-β, CTGF, and MAPK pathway regulation is dependent on PRR under HG conditions, we examined protein and mRNA levels of TGF-β and CTGF during the conditions of NG, HG, and HG plus PRO20, the pharmacological blocker of PRR. Figure 5 shows protein abundances or phospho-ERK, total-ERK, TGF-β and CTGF. The ratio of pERK/tERK vs β-actin r was increased during HG conditions and prevented by PRO20. HG alone increased protein expression of both TGF-β (2.9 ± 0.6 vs. 1.0 ± 0.3, p < 0.05) and CTGF (2.4 ± 0.3 vs. 1.0 ± 0.1, p < 0.05). In cells incubated with PRO20 before HG (HG + PRO20), upregulation of TGF-β was entirely impaired, while CTGF levels were only mitigated in comparison to HG alone (Fig. 5). These observations were seen in CTGF mRNA levels as well (NG: 0.4 ± 0.1; HG: 6.3 ± 0.8; HG + PRO20: 5.8 ± 0.9, p < 0.05) for HG and HG + PRO20. Upregulation of GLUT1 mRNA levels during HG was not prevented by PRO20 (HG: 2.1 ± 0.3 vs. 1.0 ± 0.1, p < 0.05, HG + PRO 20: 1.8 ± 0.2).

### GLUT1 inhibition but not PRR blockade prevented HG-dependent increases in intracellular ROS

**Fig. 6:**
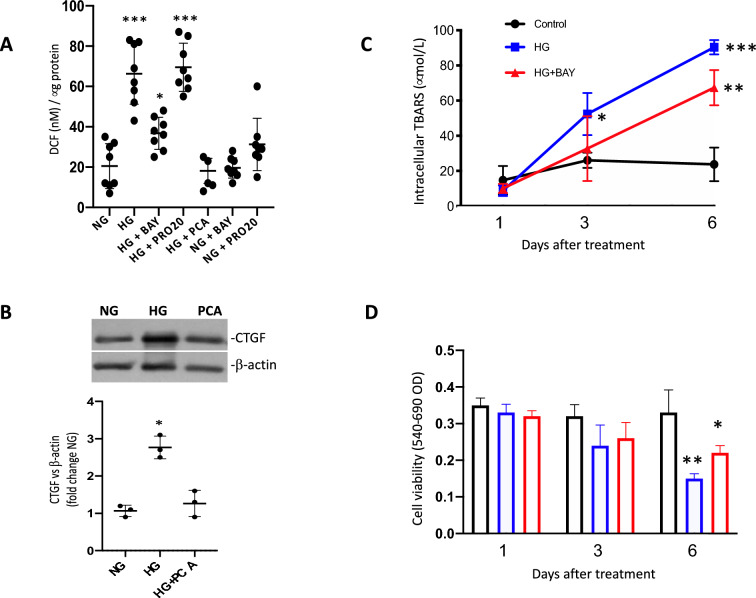
Intracellular ROS during HG conditions with or without GLUT1 blocker BAY 876, PRO20 and the effect of p-coumaric acid (PCA) on CTGF protein levels. **A** Significant increases in ROS (***p < 0.0001 vs NG, n = 8) was less evident in cells incubated with the GLUT1 specific inhibitor BAY 876 (*p < 0.05 vs NG, n = 8). PRR pharmacological blockade using PRO20 was not able to prevent the augmentation of ROS during HG (***p < 0.0001, n = 8). Co-treatment with ROS scavenger p-coumaric acid (PCA) at 10^–7^ M, was able to prevent ROS formation after 24 h HG treatment. ROS were measured by using L2',7'-dichlorodihydrofluorescein diacetate (H2DCFDA) which is intracellularly trapped by esterase activity that removes lipophilic blocking groups. Fluorescencence exhibited by dichlorofluorescien (DCF) resulting from the oxidation of the probe by ROS was normalized by micrograms of protein. **B** Representative immunoblotting showing that increased CTGF protein levels caused by HG were not observed with PCA treatment. *p < 0.05 vs NG, n = 3. **C** Intracellular TBARS was assessed in cell lysates after 1, 3 and 6 d of exposure to HG compared to controls. HG increased TBARS at day 3 and 6 while BAY876 slightly prevented this increase at day 6, although was significantly higher than cells treated in NG conditions. *p < 0.05; **p < 0.01. **D** Cell viability of cultured IMCD cells following exposure to HG during 1, 3 or 6 d demonstrated a reduced cell viability after 6 d, BAY876 (red lined bar) partially prevented this effect. Cell viability was assessed by MTT assay. *p < 0.05; **p < 0.01

Because some of the actions of PRR are mediated by ROS [30, 44] and that ROS are directly implicated in the upregulation of profibrotic markers [42, 45], we measured ROS in IMCD cells incubated for 48 h with the following conditions: (i) NG; (ii) HG, (iii) HG + BAY 876; (iv) HG + PRO20; (v) NG + BAY 876; and (vi) NG + PRO20. As shown in Fig. 6, HG alone increases intracellular ROS (67.8 ± 17 vs. control 18.1 ± 11 nM DCF/mg protein, p < 0.0001), while BAY 876 partially prevented this increase under HG conditions (33.1 ± 6 vs. control 18.1 ± 11 nM DCF/mg protein, p < 0.05). Meanwhile, PRO20 did not prevent the increases in intracellular ROS after 48 h incubations with HG (high glucose plus PRO20: 69.2 ± 12 vs. high glucose: 67.8 ± 17, p = non-significant).

### ROS scavenging prevented HG-dependent increases in CTGF

We previously demonstrated that the ROS scavenger p-coumaric acid (PCA) abolishes the prorenin-induced ROS formation and increases the expression of CTGF, α-SMA, and PAI-1 in M-1 cells [14]. Additionally, previous reports showed that the NOX-4 inhibitor GKT 137831NOX-4 inhibitor impairs ROS production and increases in fibronectin and TGF-β1 under HG conditions in vitro and in diabetic mice [19]. However, whether ROS suppression in HG conditions can prevent CTGF induction is unknown. In a new set of experiments, we performed incubations with NG, HG and HG plus a co-treatment with PCA (10 ^−7^ M) [14]. As shown in Fig. 6B, increased CTGF protein levels caused by HG were not observed with PCA co-treatment. No effect was observed in NG plus PCA (data not shown). This indicates that HG conditions promote HG-dependent upregulation of CTGF via ROS.

### Assessing time dependent cell viability versus ROS profile during HG incubations

We demonstrated that intracellular ROS was partially prevented by BAY876 incubations during 48 h of HG exposure. We also showed that PRO20, the PRR blocker, does not affect the accumulation of intracellular ROS caused by HG. To have insights into lipid peroxidation levels as an indicator of oxidative stress within the cells over the course of HG incubations, we performed a new protocol assaying lipid peroxidation in whole cell lysates. In parallel we performed a cell viability assay. For both protocols we use an n = 3 during 1, 3 and 6 d to explore the effect of chronic exposure to HG. Although BAY876 only had a mild effect on ROS we performed this experimental group to get insights about possible mechanisms. TBARS concentrations from cell lysates of experiments using 1, 3 or 6 d of HG showed that ROS increased steadily during days 3 and 6, reaching a high level at day 6 (Fig. 6B). This time course increase was slightly attenuated by BAY879 but still significantly higher than NG conditions. A reduced cell viability was found after 6 days of treatment with HG; BAY876 was not able to prevent this, although the effect was slightly reduced (Fig. 6C).

## Discussion

The PRR has become a focus of active investigation due to its particular role in enhancing intrarenal and intratubular RAS through its capacity to increase renin activity and fully activate prorenin [12, 46]. Diabetes promotes high levels of prorenin in the systemic circulation [47–49] which may contribute to the stimulating effects of prorenin on PRR in the kidney. In diabetic mice and rats, PRR is upregulated in the kidney, including the collecting ducts [4, 10, 39]. Indeed, rats that overexpress PRR show kidney damage [44] as PRR promotes intracellular pathways that increase profibrotic genes [8, 14, 31, 50]. All these factors greatly impact the development of diabetic kidney disease [13]. We recently reported that HG upregulates PRR in collecting duct cells in vivo and in vitro [8, 51]. Moreover, PRR upregulation is associated with increased expression of profibrotic markers through MAPK-dependent ROS formation in mouse renal collecting duct cells [8, 15]. However, whether glucose uptake by the collecting duct cells impacts the PRR-dependent upregulation of profibrotic factors signaling remains unclear.

Our data demonstrates: (1) HG promotes PRR upregulation in the collecting duct cells and stimulates prorenin and renin secretion to extracellular media; (2) Both effects are prevented by GLUT1 inhibition with BAY 876; (3) GLUT1-mediated upregulation of prorenin, renin, and PRR are associated with the activation of ERK1/2 and upregulation of profibrotic factors CTGF and TGF-β; (4) Specific inhibition of glucose transport via GLUT1 prevented the increases in intracellular ROS and CTGF protein levels; (5) Blockade of PRR using PRO20 does not prevent the increases in intracellular ROS and upregulation of CTGF suggesting a different pathway in the regulation of CTGF. The effectiveness of PRR blockade was confirmed by the absence of prorenin-induced phosphorylation of ERK1/2 in cells pre-treated with PRO20; (6) We observed that co-incubations with PCA, a ROS scavenger, prevented the increases in CTGF during HG conditions, suggesting that intracellular ROS is able to regulate CTGF expression and PRR activation mediated by HG-induced prorenin release can upregulate TGF-β; (7) Finally, we observed that during long-term incubations with HG (6 d) there is increased intracellular lipid peroxidation as judged by increased TBARS (Fig. 6C) which also corresponded with a decreased cell viability (Fig. 6D). Of note, in our hands, the experiments of immunofluorescence detected the colocalization of PRR and GLUT1 in the same cell, but not in AQP-2 expressing cells, suggesting that GLUT1 may be mostly present in intercalated cells in inner medullary collecting duct cells as suggested previously [26].

The kidney filters large amounts of glucose under normal physiological conditions. Glucose should be completely reabsorbed by the proximal tubule. During diabetes-induced hyperglycemia, the excessive filtration of glucose and impaired uptake by the proximal tubule promote HG delivery to the distal nephron segments [23]. Murine and rat models of diabetes and RAS activation display increased expression of GLUT1 [24]. The correlation between GLUT1 abundance and the glycolytic activity in the collecting duct supports the concept that the distal nephron segments ensure a source of energy through the glucose uptake via GLUT1 [25]. GLUT1 is abundantly expressed in principal and intercalated cells of the collecting ducts [26]. Intercalated cells also express PRR which is upregulated by HG [8, 27, 28]. We have previously reported that the intermediary Kreb´s cycle metabolite α-ketoglutarate can increase PRR expression [51] suggesting that this metabolite may be part of a metabolic pathway regulating PRR. Moreover, the special environment of the IMCD cells (e.g., hypoxia, hyperosmolality) along with the high rate of glycolysis in diabetic conditions due to higher GLUT1-mediated glucose uptake and metabolism, likely drives local accumulation of Kreb´s cycle intermediates [51, 52]. The activation of α-ketoglutarate receptor OXGR1 and succinate receptor GPR91 may be relevant in metabolic stress-related diseases such as diabetes [51, 53].

Our results suggest that the induction of ROS is mostly mediated by intracellular glucose not PRR activation, at least not at the time points assessed. HG increases the synthesis and secretion of renin and prorenin by collecting duct cells [7]. Although mostly prorenin is secreted by these cells, both renin and prorenin protein bands can be detected in IMCD and M-1 cell lysates and cell culture media when using recombinant protein standards [37]. Activation of PRR mediated by prorenin and renin increases the expression of TGF-β via a PRR-dependent activation of the MAPK pathway. This was evident by the fact that PRO20, a pharmacological inhibitor of PRR, suppressed this effect. Increases in renin synthesis, PRR upregulation, and MAPK activation may promote TGF-β expression and autocrine activation of the TGF-β receptor, which in the long term may be responsible for CTGF upregulation.

**Fig. 7:**
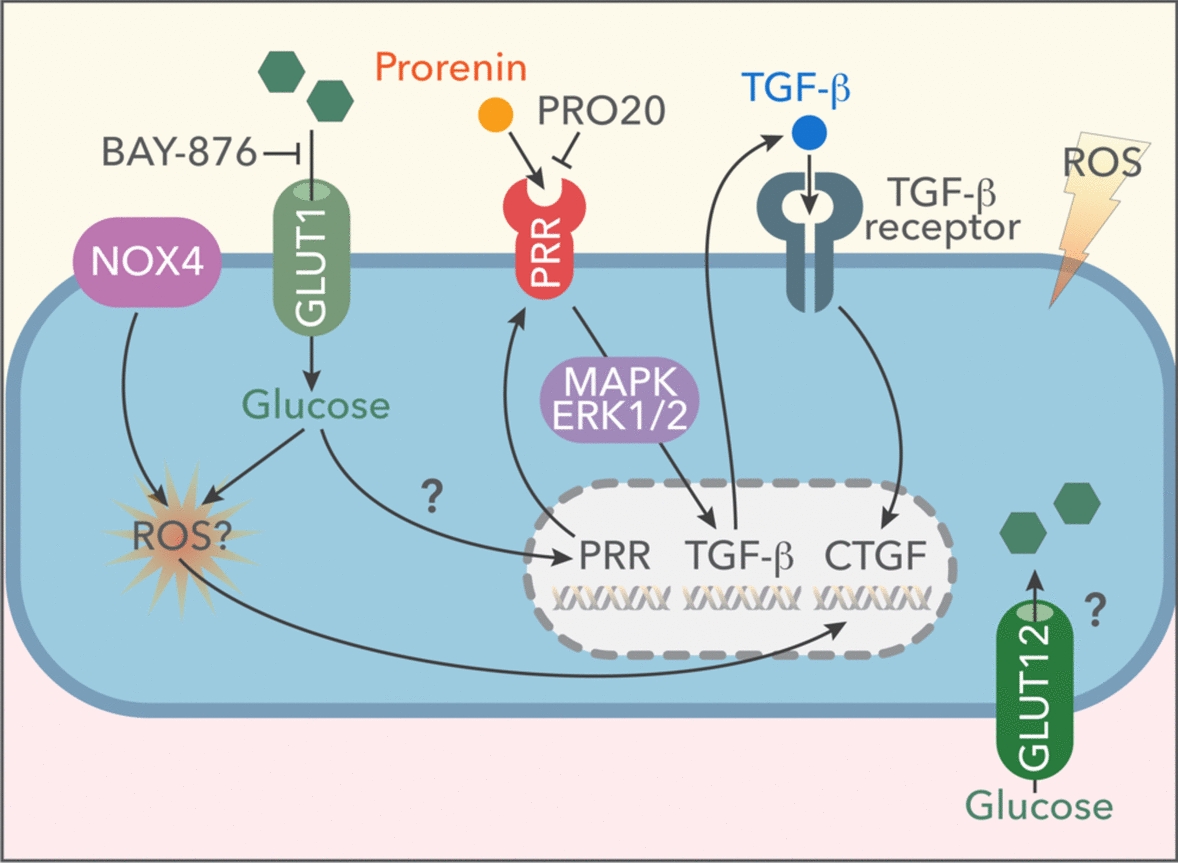
IMCD cells incubated with HG upregulate GLUT1 enabling glucose uptake. HG increases PRR synthesis along with prorenin and renin secretion in cell culture mediate. The specific GLUT1 inhibitor, BAY 876, impairs the induction of PRR, prorenin, and renin. Increases in intracellular ROS during HG conditions are attenuated by GLUT1 blockade, while the induction of TGF-β and CTGF in HG is effectively blunted by BAY876. PRO20, a PRR blocker, impairs TGF-β induction but did not prevent increases in intracellular ROS and CTGF protein expression. Treatment with ROS scavenger p-coumaric acid impairs intracellular ROS concomitant with impairment of CTGF upregulation. Intracellular ROS formation from glucose exposure or uptake may likely mediate CTGF induction which also may be sustained by TGF-β receptor in the long term. Long term effects of HG may be also related to modification of the activity of NOX4 as suggested by our previous reports. Oxidative effects of HG-dependent uptake may be also related with other suggested transporters expressed in collecting duct cells (not studied here) such as GLUT12

Here, we demonstrate that only GLUT1 blockade prevents the increases in ROS mediated by HG. It is known that ROS induces TGF-β and Smad signaling [54, 55]. Moreover, we previously showed that activation of Smad signaling causes upregulation of CTGF in IMCD cells [56, 57]. On the other hand, ERK inhibition reduces TGF-β1-stimulated Smad phosphorylation [58]. These findings may explain the differential responses by using GLUT1 inhibition and PRR blockade since PRR blockade blocks the activation of the ERK pathway responsible for TGF-β-dependent Smad regulation. Interestingly, CTGF was still augmented, despite PRR blockade. Our data suggested that ROS is responsible, at least in part, for the increases of CTGF protein expression as the augmentation was not observed when using PRR blocker PRO20. Furthermore, CTGF upregulation in HG conditions can be effectively suppressed by ROS scavenging, suggesting that CTGF depends on ROS formation (Fig. 7). Other sources of ROS may be dependent on NOX activity as demonstrated in our previous study in streptozotocin (STZ)- hyperglycemic mice [19]. Our previous reports showed that GKT 137831(NOX-4 inhibitor) impairs ROS production and increases in fibronectin and TGF-β1 in HG in vitro and in diabetic mice [19]. On the other hand, studies in cardiomyocytes demonstrated that NOX2 inhibition or knockout mice for NOX2 prevented ROS generation through the impaired O-GlcNAcylation of CaMKII and NOX2-ROS-PKC mechanism [59, 60]. Experiments on M-1 collecting duct cell lines demonstrated that NOX-4 contributes to production under HG conditions. Activation of the TGF-β1 receptor further promotes ROS production under HG conditions. Activation of the TGF-β1 receptor further promotes induction of other profibrotic factors such as CTGF and PAI-1, while fibronectin enhances fibroblast induction of other profibrotic factors such as CTGF and PAI-1, while fibronectin enhances fibroblast activation and fibroblast proliferation [15]. It is then possible that NOX4 could be activated by HG in collecting duct cells exposed to luminal and basolateral glucose. In this regard, GLUT12 expression has been shown in the cytoplasm and the apical membrane of the collecting duct in rats. Furthermore, a significant increase in GLUT12 immunolabeling can be observed in diabetic rats [24]. Interestingly, the same group has shown that induced diabetic transgenic (mRen-2, renin overexpression) rats showed increased levels of GLUT12 expression compared with non-diabetic diabetic rats. Also, long-term diabetes resulted in significant increases in GLUT1. The apical localization of GLUT12 in the distal tubules and collecting ducts suggests that it could contribute to additional glucose reabsorption in the late nephron [24].

In previous reports we demonstrated that recombinant human prorenin used at nanomolar concentrations activates MAPK pathway and upregulates cyclooxygenase-2 (COX-2) and NOX-4. This effect was also associated with the upregulation of CTGF, TGF-β, and PAI-1. These effects are blunted by pharmacological inhibition of MAPK, NOX-4, and also by the inhibition of COX-2 [15]. Furthermore, abundant expression of E-prostanoid receptors in the collecting duct supports the idea that increased COX-2 and prostaglandin formation contribute to tubular damage. In fact, the antagonism of the E-prostanoid receptor EP4, which is a Gs-coupled receptor (Gs/cAMP/PKA pathway activator), also prevented the upregulation of NOX-4 and profibrotic factors. Interestingly, the phosphorylation of Smad2/3 was prevented by EP4 antagonist, indicating that TGF-β receptor maybe not be activated due to the impairment of the autocrine actions of TGF-β [15].

Our findings could be relevant to other conditions beyond diabetes, such as hypertension. Studies in salt Dahl salt-sensitive showed the development of glomerular hypertension, which is accompanied by upregulation of TGF-β and GLUT1 which is ubiquitously expressed in mesangial cells. It has been shown that in mesangial cells cultured in vitro, overexpression of GLUT1 increases basal glucose transport, resulting in excess fibronectin and collagen production [61, 62]. These data are in accord with the results obtained in cultured collecting duct cells and in animal models of diabetes in which the upregulation of GLUT1 corelate with the increases in profibrotic factors [8, 19]. Both TGF-β and CTGF have a critical role in the development of tubule-interstitial fibrosis during diabetes [63, 64] and also in Ang II induced hypertension [65, 66]. This implicates that crosstalk between GLUT1 and intratubular RAS activity impacts tubular damage, thus both could be considered as a tissue-specific pharmacological targets. In fact, the role of GLUT1 and PRR reported in this study agrees with previous evidence showing that PRR contributes to fibrosis by the nonproteolytic activation of prorenin beyond intratubular RAS activation that leads to Ang II formation.

Clinical relevance of this study. Although the role of GLUT1 on ROS production in collecting duct cells has not been well characterized, studies in other tissues show interesting results particularly in diabetic retinopathy in which the inhibition of GLUT1 was able to reduce retinal glucose levels and glycohemoglobin levels in blood [67]. Moreover, in type 2 diabetic mice, systemic reduction of GLUT1 normalizes retinal dysfunction and Oxidative Stress [68]. The idea that GLUT1 transports more glucose when blood glucose is high, and that high glucose increases the abundance of GLUT1 agrees with previous studies [24], suggesting that GLUT1 may be a pharmacological target in kidney tissues in diabetic disease. Renal medullary transport of glucose in a Na + -independent manner was already described in 1988, demonstrating that glucose uptake into papillary collecting duct cells was mediated by a transport system similar to the one found in basal-lateral membranes [69]. Aberrant glucose uptake by cells is then an important pathophysiological mechanism underlying diabetic nephropathy in which GLUT1 may be a key player in the pathological and phenotypic changes in kidney cells during diabetic disease. Furthermore, most of the data is described in glomerular cells in which GLUT1 mediates mesangial cell glucose flux which leads to activation of signaling pathways related to glomerulosclerosis, and synthesis of TGF-β, and CTGF [70]. In mesangial cells, TGF-β activates GLUT1, leading to increased glucose flow and activation of pathways that further stimulates TGF-β synthesis, which in turn leads to amplification cycle between GLUT1 and TGF-β stimulating the progression of glomerulosclerosis, glomerular hypertension, and changes in mesangial cell phenotype. It is suggested that changes in mesangial cell phenotype activates GLUT1 and TGF-β creating an amplification cycle [71].

Limitations of the study. The present study was designed to examine the cooperative effects of GLUT1 or PRR on the regulation of profibrotic markers TGF-β and CTGF expression during HG conditions. Since we performed the study in native conditions for inner medullary collecting duct cells, we cannot rule out the effect of polarity aspects and activation of intracellular or paracrine signals such as RAS activation and the influence of Ang II as observed in other renal segments [72, 73]. However, further Ang II formation is unlikely since the endogenous formation of Ang I and AGT has not been described in IMCD cells. However, the presence of ACE in collecting duct cells may also play a role in the formation of other peptides—this possibility needs to be investigated. We have also observed that intrarenal regulation of PRR might be subjected to sex influences [74–76], thus it could be necessary to evaluate the results observed in this article in response to sex and hormonal influence in vivo and in vitro. Further studies are also required to examine the associations of glucose concentrations and temporal changes of TGF-β and CTGF expression levels.

In conclusion, we demonstrated that GLUT1 positively cooperates with the PRR-dependent upregulation of TGF-β but not CTGF which was mainly upregulated by intracellular ROS formation as judged by the results using ROS scavenging strategy in conditions of HG conditions. In addition, chronic exposure to HG in vitro causes lipid peroxidation in IMCD cells that is partially prevented by GLUT1 blockade, this suggests that ROS-mediated induction of profibrotic genes may be related to other complex mechanisms inside the cell that need further investigation.

## Materials and methods

### Animals and primary cultures of IMCD cells

Animal protocols were approved by the Institutional Review Board of the Pontificia Universidad Católica de Valparaíso (BIOEPUCV-BA 482–2022). All animal procedures were performed according to the Declaration of Helsinki and the bioethical protocol described in the approved protocol. Primary cultures of inner medullary collecting duct (IMCD) cells were obtained from 12-week-old male C57BL/6 J mice. Briefly, both kidneys were excised; inner medullary tissue was isolated and digested in solution (See Supplementary Materials). The resulting IMCD cell suspension was seeded in 3 mm Petri dishes, then divided and treated with either NG (5 mM D-glucose), HG (25 mM D-glucose), or Mannitol (25 mM) for 48 h. PRR and GLUT1 expression levels were observed to be unchanged in the mannitol-treated group (data not shown). BAY876—a highly selective and cell-permeable inhibitor of GLUT1—was used at 2 nM, added 1 h prior to NG and HG treatment, and was maintained during the next 48 h. PRO20—the PRR antagonist—was synthesized in NBC facilities and used at 1.5 μM; PRO20 was added 3 h before HG treatment and added again during the next 48 h.

### Immunoblotting analyses

Forty micrograms of protein samples were separated via electrophoresis using precast NuPAGE 10% Bis–Tris gel (Novex) at 200 v for 45 min followed by semi-dry transference to nitrocellulose membranes (Invitrogen). Blots were blocked for 3 h at room temperature, incubated with specific primary antibodies at 4 °C overnight, incubated with corresponding secondary antibodies for 45 min at room temperature, and then analyzed by normalization against the housekeeping gene β-actin. Protein level detection was performed using the antibodies listed in Table 1. The anti-PRR polyclonal antibody recognizes the intracellular segment and the ectoderm. For the MAPK pathway, we used phosphor-ERK1/2 and total ERK (Table 1). Levels of prorenin and renin in the cell culture media were assessed by Western blotting and densitometric analysis of prorenin and renin bands. 40 μg of total protein was loaded from 2 mL concentrated media using Amicon Ultra-4 Centrifugal Filter Units (Millipore). Results are presented as the ratio of protein versus β-actin in fold change of control, except for prorenin and renin bands, which were measured by intensity.

**Table 1:** Antibodies used in Western Blotting

Antibody	Manufacturer	Catalog	Target Species	Cocentration
Anti-PRR	Sigma	HPA003156	Rabbit	1:200
Anti-renin B-12	Santa Cruz Biotechnical	Sc-133145	Mouse	1:100
Anti-TGFB	Santa Cruz Biotechnical	Sc-130348	Mouse	1:500
Anti-CTGF	Santa Cruz Biotechnical	Sc-14939	Goat	1:200
Anti-phospho-p44/42 extracellular signal-regulating kinase (ERK1/2) (Thr202/Tyr204)	Cell Signaling Technology	91,065	Mouse	1:500
Anti-total extracellular regulating kinase (ERK)	Cell Signaling Technology	9122	Rabbit	1:500
Anti-beta actin	Sigma-Aldrich	A1978	Mouse	1:500

### PRR transcripts quantitation by real-time qRT-PCR

Total mRNA was isolated from mouse renal tissues using RNeasy Mini Kit (Qiagen, Valencia, CA) according to the manufacturer’s protocol. Total RNA was quantified using the nano-drop technique. Quantitative real-time PCR (qRT-PCR) was performed using the following primers: PRR: 5′-CAC AAG GGA TGT GTC GAA TG-3′, 3′-TTT GGA TGA ACT TGG GAA GC-5′; CTGF: 5′-TGCCAGTGGAGTTCAAATGC-3′; 3′-GTGTCCCTTACTTCCTGGCT-5′; TGF-β: 5′- AGAAGACGGTGTACCCCATG-3′; 5′-TGCAGTTGAGGTTCAGGACA-3′; GLUT1: 5′- GCTGTGCTTATGGGCTTCTC-3′; 5′- CACATACATGGGCACAAAGC-3′; β-actin: 5′-ATC ATG AAG TGT GAC GTT GA-3′, 3′-GAT CTT CAT GGT GCT AGG AGC-5′. Results are presented as the fold change ratio between the mRNA levels of the interest gene against the housekeeping gene β-actin in each given treatment group compared to control group.

### Immunofluorescence studies

Once cultured IMCD cells reached 70% confluence, the cells were fixed in cold methanol. They were then blocked and stained with rabbit anti-ATP6AP2 (Cat. no. HPA003156, Sigma, St. Louis, Missouri) at 1:100 dilutions, mouse anti-GLUT1 (Cat. no. PA1-46,152, Thermo-Fisher Scientific, CA) at 1:200 dilutions, and detected with Alexa Fluor 594 or 488 conjugated to antirabbit or anti-mouse IgG (Invitrogen, Carlsbad, CA), accordingly. Primary antibodies used for characterization of IMCD cells were rabbit anti-AQP-2 (Cat. no. SC-28629, Santa Cruz, CA) and rabbit anti-alpha ENaC (PA1-920A, Invitrogen, CA), both used at 1:200 dilutions. Samples were counterstained with 40,6-diamidino-2-phenylindole (DAPI, Invitrogen, Carlsbad, CA). Negative controls were obtained by omission. Immunofluorescence and phase contrast images were obtained using a Nikon Eclipse-50i immunofluorescence microscope (Nikon Eclipse-50i, Japan) and were digitalized using the NIS-Elements version 4.0 from Nikon.

### Intracellular ROS

The IMCD cells were seeded in 96-well black polystyrene plates and treated with one of the following: (i) Normal glucose (n = 8 wells); (ii) High glucose (n = 8 wells); (iii) High glucose plus BAY876 (n = 8 wells); (iv) High glucose plus PRO20 (n = 8 wells); (v) Normal glucose plus BAY876 (n = 8); or (vi) Normal glucose plus PRO20 (n = 8 wells). Then, the cells were treated with 25 μM of probe carboxy-2′, 7′-dichloro-dihydro-fluorescein diacetate (DCFHDA, Sigma Chemical Co, St. Louis, MO, USA) for 30 min at 37 °C. Fluorescence measurements of DCF (the product of H2DCFDA oxidation: excitation, 495 nm; emission, 529 nm) were performed on a plate reader (Appliskan; Thermo Fisher Scientific, Waltham, MA, USA). To normalize results, the results are shown as nM of DCF versus micrograms of protein. IMCD cells were subjected to a co-treatment with p-coumaric acid 10^−7^ mol/L (PCA; Sigma). For thiobarbituric acid reactive substance (TBARS) (lipid peroxidation) we used a colorimetric assay (Cayman Chemical, Ann Arbor, MI) according to manufacturer´s instruction. Cells were seeded and cultured under normal or high glucose with or without BAT876 and TBARS concentration of the adduct determined at 540 nm.

### Cell viability

The IMCD cells were grown in six-well plates, and after the treatments cells were washed with phosphate-buffered saline and incubated at 37 °C with 1 ml of MTT (at mg/ml during for 2 h. After replacing the MTT solution (propanol), cells were shaken at RT for 20 min. Absorbance of the samples were read at 540 and 690 nm in a spectrophotometer and results are expressed as the difference between 540 nm versus 690 nm absorbances.

### Statistical analysis

The results are expressed as the average ± the standard error. Statistical analyses were performed using GraphPad Prism Software Version 8 (GraphPad Software, Inc., La Jolla, CA, USA). Normal distribution of each parameter analyzed was tested using Shapiro–Wilk. One-way ANOVA was used to compare the mean differences between groups. When appropriate, post-test comparisons for two groups were performed using non-paired (one-tailed) Student’s *t* test. A *p*-value < 0.05 was considered statistically significant. The n number varied from 3 to 5 depending on the experiment presented.

## Supplementary Information

Additional file 1.

## Data Availability

The authors will make all methods and materials among others, available to other researchers, as requested.
