# Supplementary figures and images for: GLUT1 and prorenin receptor mediate differential regulation of TGF-β and CTGF in renal inner medullary collecting duct cells during high glucose conditions
Source: Biol Res. 2024 Nov 7;57:81. doi: 10.1186/s40659-024-00560-8 (PMC11542404; doi:10.1186/s40659-024-00560-8)

Figure S1

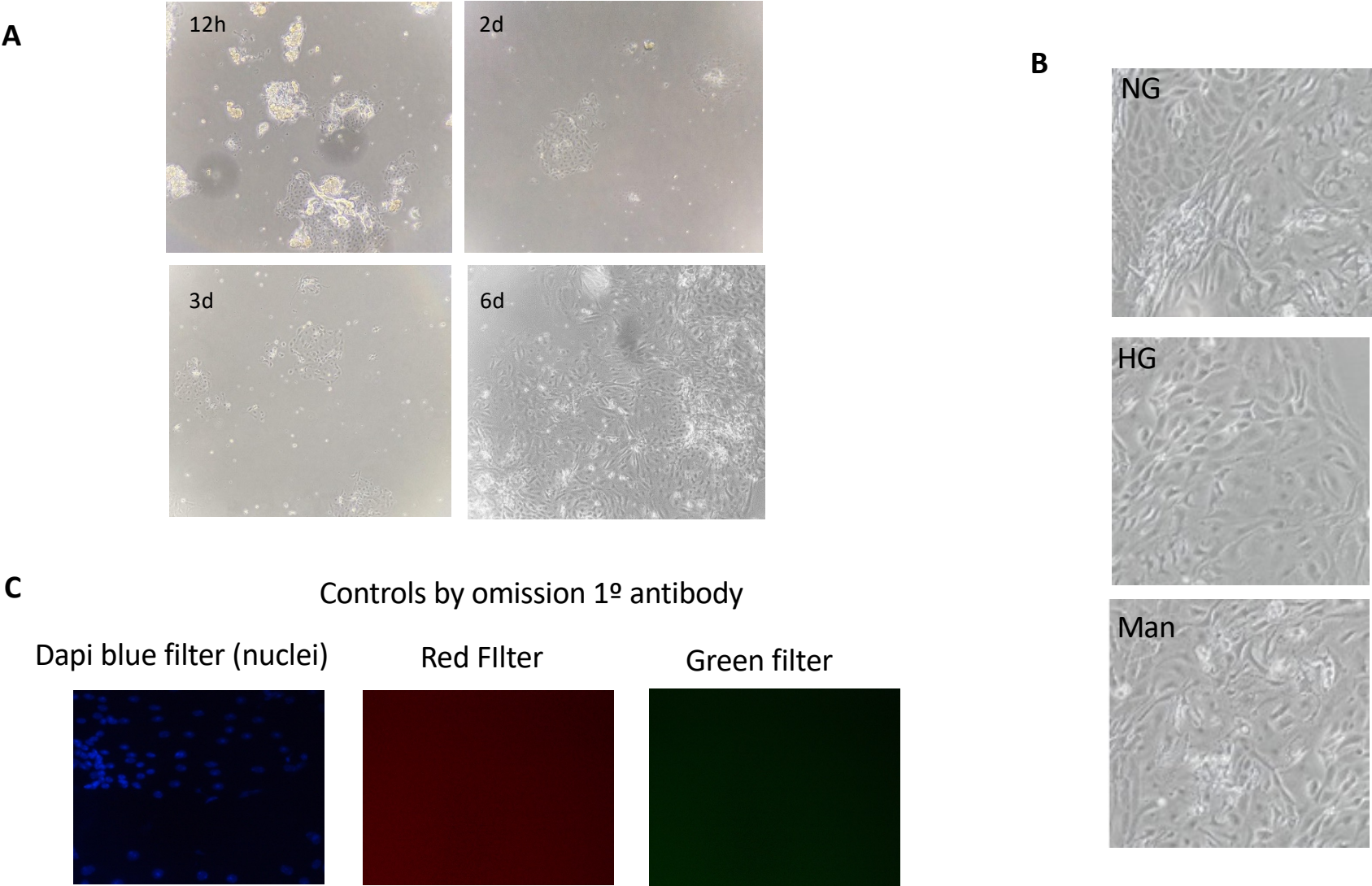

Supplement: Supplementary file 1 — Additional file 1. [file 40659_2024_560_MOESM1_ESM.pdf]
